# Emergent Inflammatory Markers and Echocardiographic Indices in Patients with Bronchial Asthma

**DOI:** 10.3390/biom13060955

**Published:** 2023-06-07

**Authors:** Nikolaos A. Gkavogiannakis, James N. Tsoporis, Ioannis-Alexandros Drosatos, George Tsirebolos, Shehla Izhar, Eleftherios Sakadakis, Andreas S. Triantafyllis, Thomas G. Parker, Lampros A. Kalogiros, Howard Leong-Poi, Loukianos S. Rallidis, Ioannis Rizos

**Affiliations:** 1 Allergy Unit “D. Kalogeromitros”, Attikon University Hospital, 124 62 Athens, Greece; nickagav@gmail.com; 2 Allergy & Clinical Immunology Department, 401 General Military Hospital of Athens, 115 27 Athens, Greece; lakalogiros@gmail.com; 3 Keenan Research Centre for Biomedical Science, Li Ka Shing Knowledge Institute, St. Michael’s Hospital, Unity Health Toronto, University of Toronto, Toronto, ON M5B 1W8, Canada; shehla.izhar@unityhealth.to (S.I.); thomas.parker@unityhealth.to (T.G.P.); howard.leong-poi@unityhealth.to (H.L.-P.); 4 Second Department of Cardiology, Attikon University Hospital, 124 62 Athens, Greece; alexdrosatos@hotmail.com (I.-A.D.); gtsirebolos@hotmail.com (G.T.); elsakadakis@yahoo.gr (E.S.); andtridoc@yahoo.gr (A.S.T.); lrallidis@gmail.com (L.S.R.); ioannis.c.rizos@otenet.gr (I.R.); 5 Department of Cardiology, 414 Military Hospital, P. Penteli, 152 36 Athens, Greece; 6 Department of Cardiology, 401 General Military Hospital of Athens, 115 27 Athens, Greece

**Keywords:** asthma, oxidative stress, inflammation, sRAGE, S100A12, IL-6, DJ-1

## Abstract

Asthma is a heterogeneous disease, characterized by chronic inflammation and oxidative stress of the airways. Several inflammatory pathways including activation of the receptor for advanced glycation end products (RAGE) have been described in the course of the disease. DJ-1 is a redox-sensitive protein with multifaceted roles in mast cell homeostasis and an emerging role in the pathogenesis of asthma. Moreover, cardiac function abnormalities have been described via echocardiography in patients with asthma. The main aim of this study was to investigate the plasma levels of RAGE, its ligands and DJ-1 in asthmatic patients pre- and post-treatment along with echocardiographic indices of cardiovascular function. The study population was divided into two groups. Group A included 13 patients with newly diagnosed bronchial asthma who were free of treatment for at least two weeks and Group B included 12 patients without asthma. An echocardiography examination was performed on all patients. The plasma levels of RAGE, its ligands (AGEs, S100A12, S100B, S100A8/A9), the interleukins (IL-6, IL-1β) and DJ-1 were measured. No differences were noted among the two groups for baseline characteristics and echocardiographic indices of cardiac function. In Group A, 31% suffered from mild asthma, 54% from moderate asthma and 15% from severe asthma. Plasma levels of IL-6, AGEs and AGE/RAGE ratio were increased and those of S100A12 and DJ-1 were decreased in asthmatics. Pharmacotherapy with corticosteroids/β2-agonists decreased IL-6, and AGEs, and increased DJ-1. In search of novel approaches in diagnosing and treating patients with asthma, S100A12, ratio AGE/sRAGE, and DJ-1 in addition to IL-6 may prove to be useful tools.

## 1. Introduction

Asthma is one of the major noncommunicable diseases, affecting 1–18% of the population globally [1]. It is characterized by variable airflow obstruction and respiratory symptoms as dyspnea, wheezing, chest tightness and cough. The interplay between genetic factors and environmental exposures in asthmatic patients results in a chronic, heterogeneous inflammation of the airways. Moreover, in more than half of patients with asthma, allergy has a prominent role and exposure to exogenous stimulants, as pollen, mite, animal, or fungal allergens play a crucial role in the pathogenesis [2]. In most asthmatics, T2^high^ inflammation is prominent, and IL-4, IL-5, IL-9 and IL-13 play a major role. There are, however, patients with asthma characterized by non-T2 inflammation pathways, driven by IL-6, IL-1β, and IL23 [3]. In addition to inflammation, reactive oxygen species (ROS), endogenous nitric oxide (NO) and NO-derived reactive nitrogen species (RNS) have been reported to mediate oxidative stress and have an important role in airway inflammation and the pathogenesis of asthma [4].

The receptor for advanced glycation end products (RAGE) is a member of the immunoglobulin superfamily of cell surface receptors acting as a pattern recognition receptor [5]. RAGE ligands include the S100/calgranulin proteins, advanced glycation end products (AGEs), Mac-1, high mobility group box-1, amyloid-β peptide, β-sheet fibrils, lysophosphatidic acid, and the redox-sensitive protein DJ-1 [6,7,8,9]. RAGE-ligand binding upregulates proinflammatory pathways, via signaling through NFkB [6,7,8]. RAGE expression is highest in the lungs and has been implicated as a driving force for inflammation in pulmonary pathophysiology [10]. RAGE contributes to several lung diseases including asthma. The soluble isoform of RAGE (sRAGE) acts as a ‘decoy’ to sequester RAGE ligands, and thus prevents their binding to the receptor [5]. Several reports have linked deficiency of sRAGE to the severity and outcomes of various human diseases, and association with RAGE G82S variants [11]. However, to date, there have been few studies that have examined the association between sRAGE and asthma. These studies had small sample sizes and their results were contradictory [12,13,14]. For example, Sukkar et al. [13] reported that sRAGE was deficient in neutrophilic asthma. Other studies, however, reported elevated levels of sRAGE in asthmatics [12,14]. The potential reason for the discrepancy may be attributable to the different asthma inflammatory phenotypes, which are classified as neutrophilic and non-neutrophilic based on induced sputum inflammatory cells [13] and are associated with RAGE genetic variants. With respect to the RAGE ligands and asthma, there is scarce data available. 

There are few reports investigating the relationship between asthma and heart disease. Prospective epidemiological studies have reported higher cardiovascular event rates in asthma patients than those without asthma [15,16]. There seems to be no correlation between heart disease and asthma [15], and transthoracic echocardiogram findings are scarce in patients with asthma. 

Biomarkers are increasingly recognized to have significant clinical value in the early identification and progression of various cardiovascular diseases. There are many heart conditions, such as congestive heart failure, ischemic heart diseases, diabetic cardiomyopathy, and cardiac remodeling, in which the severity of the cardiac pathology can be mirrored through these cardiac biomarkers [17,18,19]. Different biomarkers of heart failure may reflect the underlying mechanisms/pathways of heart failure and its progression and point out specific therapy options. Alternatively, these biomarkers may reflect coexisting or isolated disease processes in different organ systems other than the cardiovascular system. Although levels of RAGE ligands S100B, S100A8/A9, S100A1, S100A6, AGEs may have a biomarker role in cardiovascular disease [17,18,19], in asthma, there are scarce data advocating such a role. 

The receptor for advanced glycation end products (RAGE) is expressed in the heart in cardiomyocytes, vascular cells, fibroblasts, and infiltrating inflammatory cells [5]. Experiments in murine, rat, and swine models of injury suggest that RAGE and the ligands of RAGE are upregulated in key injuries to the heart, including ischemia/reperfusion injury, diabetes, and inflammation [5]. Pharmacological antagonism of RAGE or genetic deletion of the receptor in mice is strikingly protective in models of these stresses [18]. Data emerging from human studies suggests that measurement of levels of RAGE ligands or soluble RAGEs in plasma or serum may correlate with the degree of heart failure [17,18,19]. Taken together, the ligand–RAGE axis is implicated in heart failure, and the therapeutic antagonism of RAGE might be a unique target for therapeutic intervention in this disorder.

Although various therapeutic agents have been developed for asthma and heart failure, respectively, therapeutic drugs for each disease may adversely affect another disease (e.g., β-blockers for heart failure and β-agonists for asthma). We conducted a prospective observational study to clarify whether the coexistence of asthma is a poor prognostic factor for heart-related disease. The aim of this study is to investigate the plasma expression of novel (sRAGE, RAGE ligands, and DJ-1) in comparison to more common (IL-6, IL-1β) inflammatory markers in asthmatic patients with different inflammatory phenotypes, and the cardiac impact through heart ultrasound findings. 

## 2. Methods

### 2.1. Participants

A total of 25 subjects, 13 with newly diagnosed asthma and 12 non-asthmatic subjects (healthy control), were included in this study (Table 1). Asthmatic subjects were recruited according to the following criteria: (1) history of symptoms related to asthma such as cough, wheezing, dyspnea or chest tightness (2) obstruction in spirometry and a positive bronchodilator response after inhalation of 400 mg salbutamol, which defined as 200 mL and 12% increase in forced expiratory volume in 1 s (FEV1″). According to their symptoms, restriction in quality of life and spirometry results, asthma was characterized as mild, moderate, or severe, and inhalation of corticosteroids (ICS) plus long-acting beta agonist (LABA) was prescribed for 12 weeks to severe patients, according to global initiative for asthma (GINA) guidelines. Exclusion criteria were as follows: (1) any asthma treatment during the previous 2 weeks; (2) severe asthma exacerbation in the previous 2 months; (3) body mass index (BMI) above 30; (4) arterial hypertension; (5) diabetes mellitus; (6) smoking or alcohol abuse; (7) pregnancy or lactation; (8) respiratory/heart/kidney/liver failure, (9) heart valve disease; (10) cancer; (11) current infection; and (12) autoimmune disease. The study protocol was approved by the Ethic committee of “Attikon” University General Hospital of Athens and written informed consent to the study was obtained from all participants.

### 2.2. Intervention

Medical history, in particular respiratory symptoms and asthma control test, and use of smoking and alcohol were ascertained by a questionnaire. Smoking and alcohol use were classified as current habitual use or not. Height and weight were measured, and BMI (BMI: kilograms per meter squared) was calculated as an index of the presence or absence of obesity. Blood pressure (BP) was measured in the sitting position using an upright standard sphygmomanometer. Spirometry was performed with a Viasys JAEGER type APS-Pro spirometer and post-bronchodilation values were measured after inhalation of 400 mg salbutamol (Aerolin^®^ recording). Exhaled nitric oxide was measured with the NO CLD88 SP-ECOMEDICS device. Skin prick testing was performed to the most relevant aeroallergens according to the GA2 LEN study [20]. This panel includes the following 18 allergens: hazel (Corylus avellana), alder (Alnus incana), birch (Betula alba), plane (Platanus vulgaris), cypress (Cupressus sempervirens), grass mix (smooth meadow grass/Poa pratensis, cock’s foot grass/Dactilis glomerata, perennial rye grass/Lolium perenne, timothy grass/Phleum pratense, meadow fescue/Festuca pratensis, meadow oat grass/Helictotrichon pretense), Olive (Olea europaea), mugwort (Artemisia vulgaris), ragweed (Ambrosia artemisiifolia), Alternaria alternata (tenuis), Cladosporium herbarum, Aspergillus fumigatus, Parietaria, cat, dog, Dermatophagoides pteronyssinus, Dermatophagoides farinae, and cockroach (Blatella germanica). An electrocardiogram and transthoracic echocardiogram were also performed on each patient at the same visit by an associate cardiologist at Attikon University General Hospital of Athens, focus on the following data: ejection fraction, right atrial volume, right ventricular volume, and pulmonary artery systolic pressure.

### 2.3. Blood Samples

Blood was drawn from the antecubital vein in the morning for determining common blood count, erythrocyte sedimentation rate, lipids (total cholesterol, LDL cholesterol, triglycerides, and HDL cholesterol), plasma glucose, HbA1c, creatinine, urea, aspartate aminotransferase (AST), and alanine aminotransferase (ALT), total serum IgE and specific IgE for the positive skin prick test. The above-mentioned parameters were evaluated at a commercial laboratory. Blood samples were drawn in tubes without additives, containing heparin or citrate (for routine biochemistry).

For AGE, S100B, S100A12, S100A8/A9, sRAGE, DJ-1, IL-6, and IL-1β determination, blood samples were collected in tubes containing EDTA, centrifuged at 4 °C and immediately divided into aliquots. Plasma samples were stored at −70 °C until analysis. The plasma concentrations of S100A8/S100A9, sRAGE, DJ-1, S100B, IL-6, IL-1β and S100A12 were quantified using the human S100A8/S100A9 heterodimer (sensitivity—21.5 pg/mL), sRAGE (sensitivity—16.14 pg/mL), DJ-1/Park7 (sensitivity—62.5 pg/mL), S100B (sensitivity—50 pg/mL), IL-6 (sensitivity—6.4 pg/mL), IL-1β (sensitivity—1 pg/mL), S100A12 (sensitivity—7.8 pg/mL) DuoSet ELISA kits, respectively, together with the DuoSet ELISA Ancillary Reagent Kit 2 according to the manufacturer’s instructions (R&D Systems Inc., Minneapolis, MN). The plasma concentration of AGE (Advanced Glycation End) was quantified with the Assay Kit (sensitivity—0.5 mg/mL) (ab238539, abcam, Cambridge, UK). All samples were processed blindly.

### 2.4. Statistical Analysis

Normally distributed variables are expressed as mean and standard deviation. Non-normally distributed data are presented as median and inter-quartile range (IQR). Differences in continuous variables were estimated using *t*-test or the Mann–Whitney U-test as appropriate. Differences in categorical data were evaluated via χ^2^ or Fisher’s exact test. Limitations regarding the small number of participants were the main reason we performed correlations, rather than regression analysis. Correlations were estimated using Spearman’s correlation method for nonparametric variables and Pearson correlation for numerical variables. Values of *p* < 0.05 were considered statistically significant.

## 3. Results

### 3.1. Baseline Clinical and Laboratory Characteristics

A total of 13 asthmatic patients were included in this study, with a mean age of 36 years. Of the cohort, nine (69%) were allergic, with at least one positive skin prick test to aeroallergens. Demographic characteristics of the population included in our study are shown in Table 1. Patients with asthma were mostly male, had decreased levels of asthma control test, and a trend (N.S.) towards increased levels of total IgE, compared to controls.

All study groups were comparable with respect to age, BMI, and levels of common biochemistry measures and CRP. All the asthmatic patients had a positive bronchodilator response after 400 mg salbutamol with a mean increase in FEV1′ of 370 mL (12.4%) vs. 180 mL (0.5%) in control group.

Furthermore, patients with asthma had increased mean levels of fractional exhaled nitric oxide (FeNO) in comparison to controls (90.4 ppb vs. 28 ppb, respectively). FeNO was used as a surrogate biomarker for type 2 inflammation, and the data suggest that patients with higher FeNO levels respond better to inhaled corticosteroid (ICS) treatment [21]. Allergy status or asthma severity had no impact on measured inflammatory/oxidative markers, except for IL-6, which was higher in the allergic individuals with asthma vs. control (26.08 pg/mL vs. 9.92 pg/mL, *p* = 0.043).

### 3.2. Cardiac Findings (Electrocardiogram and Echocardiogram)

All subjects (control and asthmatic) were in sinus rhythm with normal left ventricular ejection fraction (LVEF > 50%) calculated according to the modified Simpson’s method [22]. Asthmatics had no cardiac abnormalities, and transthoracic echocardiogram measurements were within the range observed in controls (Table 1). Both controls and asthmatics had normal left ventricle dimensions and Doppler measurements (Table 1). Interestingly, we observed a trend for higher (but not abnormal) levels of pulmonary systolic artery pressure in patients with asthma.

### 3.3. Serum Levels of IL-6, IL-1β, RAGE Ligands and DJ-1

**Figure 1:**
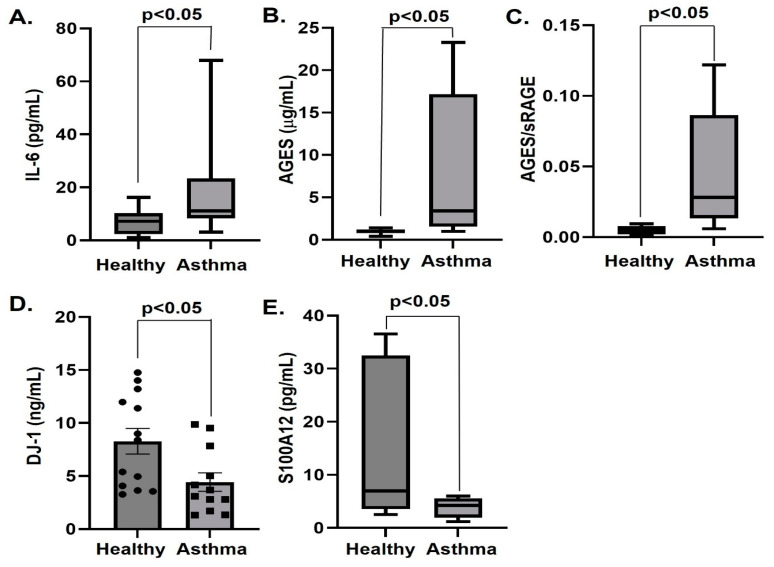
Plasma levels of IL-6 (pg/mL) (**A**), AGEs (μg/mL) (**B**), AGEs/sRAGE (**C**), DJ-1 (ng/mL) (**D**) and S100A12 (pg/mL) (**E**) in healthy controls and asthmatics presented as box plots showing median and interquartile range or mean ± SEM (DJ-1). *n* = 12–13.

**Figure 2:**
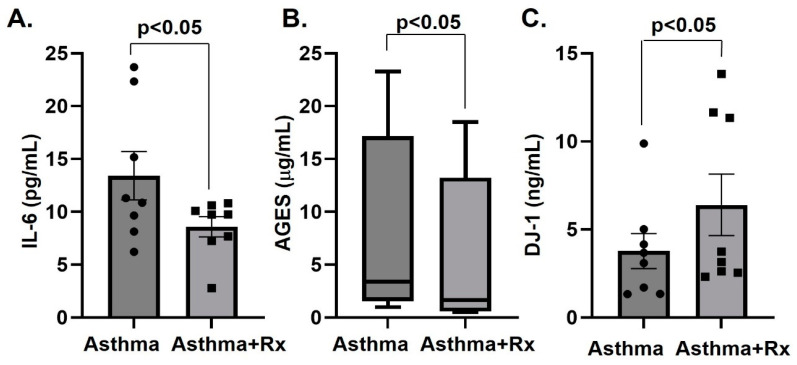
Plasma levels of IL-6 (pg/mL) (**A**), AGEs (mg/mL) (**B**), DJ-1 (ng/mL) (**C**) in asthmatic patients pre- and post-pharmacotherapy (Rx) with corticosteroids and/or β2 agonists presented as mean ± SEM (Il-6, DJ-1) or box plot showing median and interquartile range (AGEs). *n* = 8.

The levels of IL-6, IL-1β, sRAGE, AGEs, S100B, S100A8/A9, S100A12(EN-RAGE) and DJ-1 together with the biomarker for disease ratio AGEs/sRAGE [23] were determined in asthmatic patients and control subjects. The plasma levels of IL-1β, S100B, S100A8/A9 and sRAGE were not altered in asthmatics (Table 2). Compared to the control group, asthmatics had increased plasma levels of the inflammatory cytokine IL-6, AGEs, and of the AGE/sRAGE ratio (irrespective of unchanged sRAGE) (Table 2, Figure 1). In contrast, plasma concentrations of DJ-1 and S100A12 were decreased in asthmatics compared to controls (Table 2, Figure 1). Pharmacotherapy (inhalation of corticosteroids plus long-acting beta agonist according to the global initiative for asthma (GINA) guidelines) prescribed to a subgroup (*n* = 8) of the asthmatic patients with severe asthma for 12 weeks decreased IL-6 and AGE, and increased DJ-1 (Table 3, Figure 2).

### 3.4. Correlations of Inflammatory Markers and Spirometry Parameters

We observed significant (*p* < 0.05) correlations between S100B, S100A8A9, DJ-1, IL-6, and post-treatment levels of FEV1″, FEV1″/FVC, and MEF_75–25_. Negative correlations were obtained between S100B and post-treatment (post-Rx) FEV1″/FVC (the proportion of a person’s vital capacity that they are able to expire in the first second of forced expiration to the full, forced vital capacity), S100B and post-Rx MEF_75–25_ (average flow rate between 25% and 75% of the exhaled volume), S100A8A9 and post-Rx FEV1″/FVC, and S100A8/A9 and post-Rx MEF_75–25_ (Table 4). Positive correlations were observed between DJ-1 and post-Rx MEF_75–25_, IL-6 and post-Rx FEV1″, and DJ-1 and sRAGE (Table 4).

## 4. Discussion

To the best of our knowledge, this is the first study to evaluate biomarkers of inflammation and oxidative stress in patients with asthma which do not have cardiac abnormalities as quantified via normal findings on electrocardiogram and transthoracic echocardiogram.

In this study, allergic asthmatic patients with elevated T2 inflammation markers (high FeNO levels) and controls were screened via echocardiography and plasma inflammatory markers were assessed and compared to controls. Apart from a modest increase in pulmonary artery systolic pressure in asthmatics, there were no differences between the two groups regarding cardiac findings.

To better understand the inflammatory/oxidative response in asthmatics, we quantified the plasma levels of a novel set of markers the RAGE ligands and the antioxidant DJ-1. The diverse markers assessed provide us with novel opportunities in understanding the inflammatory/oxidative response(s) in patients with asthma. RAGE ligands that prolong the inflammatory response through internal pathways and DJ-1 may play a key role in the pathophysiology of asthma.

A wide range of clinical and experimental studies have found that RAGE plays a significant inflammatory role in the pathogenesis of allergic airway disease [24,25]. RAGE has been identified as a pattern recognition receptor and a danger signal (or alarmin) receptor [5]. In addition to binding AGEs, RAGE also binds a multitude of other non-glycated ligands, the majority of which are damage-associated molecular patterns (DAMPs) such as the S100 family. In our study, we showed that the AGEs and, importantly, the ratio AGE/sRAGEs were increased in asthmatic patients and this increase was attenuated in the presence of pharmacotherapy with inhalation of corticosteroids plus long-acting beta agonists. There was a tendency for a decreased sRAGE in asthmatics; however, it did not reach statistical significance, which many relate to the small sample size. Overall, the trends indicate that sRAGE levels are decreased in asthmatic subjects, which was inversely correlated with disease severity [11,13,26,27]. Since sRAGE binds RAGE ligands, dampening the robust inflammatory responses perpetuated through its feed-forward signaling [28], lower sRAGE levels may augment development and/or progression of asthma. Studies are needed to determine any differences in overall expression levels and cellular localization of RAGE and sRAGE in asthmatics compared to non-asthmatics. With respect to RAGE ligand levels, we showed no change in S100B, and S100A8/A9 but decreased S100A12 in asthmatics compared to healthy controls. This is the first study that showed S100B levels are unaltered in asthmatics compared to controls. In contrast to our finding, several studies have reported enhanced S100A8 and S100A9 in asthmatics and show a strong correlation with disease severity [29,30], whereas one study found S100A8 and S100A9 to be decreased in the sputum of mild asthmatics with no evidence of increased inflammation compared to control subjects [31]. Interestingly, the administration of neutralizing antibodies to S100A8 or S100A9 as potential therapeutic strategy had no effect on airway hyper-responsiveness, had minimal effect on lung tissue inflammation, and reduced eosinophil or neutrophil cell counts in BALF only slightly [32,33]. However, we report decreased plasma levels of S100A12 independent of pharmacotherapy in asthmatics compared to controls; in addition, elevated S100A12 has been reported in the in the BALF of asthmatic patients after experimental allergen exposure and in the sputum of patients with neutrophilic or eosinophilic asthma compared to healthy controls [31,34]. However, in line with our data showing diminished S100A12 in the plasma of asthmatics, transgenic expression of human S100A12 in pulmonary smooth muscle of mice resulted in attenuated native and allergen-induced airway responsiveness and exhibit enlarged airways with less airway smooth muscle compared to wild type mice [35]. Thus, therapeutic administration of S100A12 protein to the airway smooth muscle could be envisioned to reduce muscle mass and, consequently, improve chronic lung inflammation and constrictor responsiveness as seen in asthmatics.

Asthma has been reported to be associated with oxidative stress [36]; yet, no function has been directly attributed to DJ-1. We measured plasma levels of DJ-1 and showed asthmatics had lower DJ-1 levels compared to healthy controls, and a positive response to DJ-1 after treatment with inhaled corticosteroids and/or β2-agonists. Our results agree with a previous study showing markedly diminished serum levels of DJ-1 with corresponding increases in ROS levels in asthmatics when compared to normal subjects [37]. DJ-1 acts as an antioxidant that is capable of quenching ROS production [38,39]. In asthma, production of ROS accompanies mast cell activation is thought to promote this activation [36,40]. However, the extent to and mechanisms through which DJ-1 could regulate ROS in these activities is unknown.

The pro-inflammatory cytokine IL-6 was increased in patients versus control and 3 months post-pharmacotherapy, the levels were decreased. The pleiotropic role of IL-6 [41], which promotes Th2 and Th17 differentiation, plays a regulatory role for asthma and in our study, which has mostly patients with an allergic status (T2 inflammation), it seems to be concordant.

The plasma levels of AGE and ratio of AGE/sRAGE were higher in asthmatic patients irrespective of unchanged plasma sRAGE as compared to control subjects. Ratio AGE/sRAGE depicts a surrogate biomarker which can be used for asthma diagnosis, and subsequent management and treatment which affect the ratio could demonstrate benefits for asthmatics.

Following treatment of asthma with inhaled corticosteroids and long-acting β_2_-agonists, we observed a decrease in IL-6 and AGEs and an increase in DJ-1 levels. Moreover, DJ-1 positively correlated with small airway disease as depicted by post-Rx MEF_75–25._ Furthermore, S100B and S100A8/A9 inversely correlated with post-Rx Tiffenaeu (FEV1″/FVC) and post-Rx MEF_75–25_, suggesting that asthma is under control inflammation wanes. An unexpected finding was the positive correlation of IL-6 with post-FEV1″; however, it may reflect the better response to treatment in patients with higher burden of inflammation.

This study has certain strengths and limitations. The diversity of the measured inflammatory markers (AGEs, sRAGE, S100 proteins, DJ-1, interleukins) together with the measurement of echo-derived parameters of cardiac function can be marked as strengths of the present study. Several limitations should also be acknowledged. The relatively small number of participants as a first diagnosis of asthma was a prerequisite and there are limitations inherent to the cross-sectional, observational design, as we evaluated association, not prospective prediction or causation. The ELISA detection system used in the present study could measure only total sRAGE levels and cannot discriminate between specific soluble RAGE variants (cRAGE and esRAGE); therefore, decreased sRAGE levels as determined by this assay may be caused by a decrease in distinct circulating sRAGE isoforms. We had to exclude inflammatory markers with very low to undetectable plasma concentrations. Nonetheless, the selected biomarkers have completeness of measurements, indicating acceptable quality of quantification. Our population is <40 years. Therefore, generalization of the results to an older age category should be carried out with caution. Our study only indicates an association; we believe that further studies are needed to establish the causal role of DJ-1, RAGE, and its ligands in the pathogenesis of asthma.

Despite these limitations, the findings discussed here indicate that the AGEs-RAGE system and DJ-1 are closely related with the pathophysiology of asthma, and AGEs/sRAGE ratio, S100A12 and DJ-1 could serve as novel biomarkers to predict asthma treatment. It is tempting to speculate that, in the upcoming years, targeting S100A12 and DJ-1 may be an effective viable therapy in patients with severe, persistent asthma.

## 5. Conclusions

In conclusion, we suggest that the plasma measurement of IL-6, AGE/sRAGE ratio, S100A12 and DJ-1 provides a promising and non-invasive technique that facilitates the detection of inflammation and measurement of therapeutic efficacy in asthmatics. However, long-term prospective studies are warranted to elucidate their role in asthma and to investigate whether modulation of the circulating levels of pro-inflammatory/antioxidant proteins can modify disease severity.

## Figures and Tables

**Table 1:** Patient characteristics.

	Healthy	Asthmatics	*p*
***n***	12	13	N.S.
Age (years)	35 ± 3.5	37 + 5.8	N.S.
Sex (Female)	4	5	N.S.
BMI (Kg/m^2^)	22.1 ± 6.4	24.33 ± 3.03	N.S.
E (m/s)	0.86 ± 0.15	0.77 ± 0.13	N.S.
A (m/s)	0.45 (0.44, 0.46)	0.51 (0.48, 0.56)	N.S.
E/A	1.7 ± 0.3	1.5 ± 0.2	N.S.
RVD(4CHmid) (mm)	29.3 ± 2.5	30.1 ± 4.2	N.S.
LVESD (mm)	44.3 ± 3.2	45.3 ± 5.6	N.S.
LVEDD (mm)	26.7 ± 2.1	30.1 ± 4.1	N.S.
Left atrium (mL)	32.1 ± 2.1	33.5 ± 4.3	N.S.
PASP (mmHg)	14.2 ± 2.8	19.1 ± 6.2	N.S.
Right atrium (mL)	27.6 ± 5.8	30.75 ± 6.57	N.S.
SRV (cm/s)	14.3 ± 2.5	13.8 ± 2.1	N.S.
TAPSE (mm)	26.7 ± 2.7	24.3 ± 2.8	N.S.
tIgE (IU/L)	155.2 ± 61.4	173.4 ± 73.2	N.S.
FeNO (ppb)	0 (0, 30.5)	19.7 (0, 134.9)	N.S.
FEV1″ (L)	4.0 ± 0.8	3.3 ± 0.6	N.S.
FEV1″ (%)	99.5 ± 9.9	96.0 ± 11.4	N.S.
FVC (L)	5.2 ± 1.1	4.7 ± 0.9	N.S.
FVC (%)	108.0 ± 7.2	113.4 ± 14.3	N.S.
MEF (%)	75.7 ± 28.6	55.8 ± 11.1	N.S.
EF (%)	65.1 ± 3.5	62.8 ± 3.6	N.S.

BMI—Body mass index; E—early diastolic velocity; A—atrial velocity; RDV(4CHmid)—right ventricle diameter (4 chambers mid); LVESD—left ventricle end systolic diameter; LVEDD—left ventricle end diastolic diameter; PASP—pulmonary artery systolic pressure; SRV—systolic excursion velocity of right ventricle; TAPSE—tricuspid annular plane systolic excursion; FeNO—fractional exhaled nitric oxide; tIgE—serum total IgE, FEV1″—forced exhaled in 1″; FVC—forced vital capacity, FVC—forced vital capacity; MEF—mid-expiratory flow; EF—ejection fraction.

**Table 2:** RAGE ligands, soluble (s)RAGE and DJ-1 in plasma of healthy controls and asthmatics.

	Healthy	Asthmatics	*p*
***n***	12	13	
DJ-1 (ng/mL)	8.4 (3.9, 12.6)	3.4 (1.9, 7.1)	<0.05
S100A12 (ng/mL)	6.9 (3.6, 32.5)	4.3 (1.9, 5.6)	<0.05
IL-1β (pg/mL)	5.1 (3.1, 49.4)	4.1 (3.1, 8.4)	N.S.
IL-6 (pg/mL)	7.2 (2.4, 10.3)	11.1 (8.3, 23.4)	<0.05
AGE (μg/mL)	1.0 (0.8, 1.2)	3.4 (1.6, 17.2)	<0.05
Ratio AGE/sRAGE (%)	0.005 (0.002, 0.008)	0.028 (0.013, 0.086)	<0.05
S100A8/A9 (ng/mL)	1324.7 (171.8, 1884.2)	559.1 (288.3, 1599.7)	N.S.
S100B (pg/mL)	181.1 ± 38.6	216.7 ± 135.9	N.S.
sRAGE (pg/mL)	209.8 (120.7, 621.5)	156.4 (94.2, 304.7)	N.S.

AGE—advanced glycation end products; sRAGE—soluble receptor for advanced glycation end products.

**Table 3:** RAGE ligands, soluble (s)RAGE and DJ-1 in plasma of asthmatics.

	Pre-Rx	Post-Rx	*p*
***n***	8	8	
DJ-1 (ng/mL)	3.8 + 0.9	6.4 + 1.7	<0.05
S100A12 (ng/mL)	5.2 (2.4, 5.6)	2.4 (1.9, 6.0)	N.S.
IL-1β (pg/mL)	3.9 (3.1, 7.3)	3.2 (2.9, 6.1)	N.S.
IL-6 (pg/mL)	13.4 + 2.3	8.6 + 2.7	<0.05
AGE (μg/mL)	3.4 (1.6, 17.2)	1.7 (0.6, 13.2)	<0.05
Ratio AGE/sRAGE (%)	0.044 + 0.015	0.036 + 0.017	N.S.
S100A8/A9 (ng/mL)	2362.9 + 1294.2	3654.6 + 1793.3	N.S.
S100B (pg/mL)	225.4 + 56.7	150.7 + 36.2	N.S.
sRAGE (pg/mL)	189.7 + 58.4	170.1 + 46.4	N.S.

AGE—advanced glycation end products; sRAGE—soluble receptor advanced glycation end products.

**Table 4:** List of correlations.

	FEV1″(Post Rx) r	*p*	FEV1″/FVC (Post Rx) r	*p*	MEF75–25 (Post Rx) r	*p*	sRAGE r	*p*
**S100B**					−0.737	<0.05		
S100A8/A9			−0.762	<0.05	−0.712	<0.05	0.403	<0.05
DJ-1			−0.787	<0.05	0.833	<0.05		
IL-6	0.714	<0.05						

Abbreviations: post Rx: post treatment, r: correlation coefficient, FEV1″/FVC: (proportion of a person’s vital capacity that they can expire in the first second of forced expiration to the full, forced vital capacity), MEF75–25: (average flow rate between 25% and 75% of the exhaled volume).

## Data Availability

All available data from this study appears in this manuscript.
